# Loss of type 9 adenylyl cyclase triggers reduced phosphorylation of Hsp20 and diastolic dysfunction

**DOI:** 10.1038/s41598-017-05816-w

**Published:** 2017-07-17

**Authors:** Yong Li, Tanya A. Baldwin, Yan Wang, Janani Subramaniam, Anibal Garza Carbajal, Cameron S. Brand, Shane R. Cunha, Carmen W. Dessauer

**Affiliations:** 1 Dept. Integrative Biology and Pharmacology, McGovern Medical School, University of Texas Health Science Center, Houston, TX 77030 USA; 2 Department of Pharmacology, School of Medicine, University of California San Diego, La Jolla, CA 92093 USA

## Abstract

Adenylyl cyclase type 9 (AC9) is found tightly associated with the scaffolding protein Yotiao and the I_Ks_ ion channel in heart. But apart from potential I_Ks_ regulation, physiological roles for AC9 are unknown. We show that loss of AC9 in mice reduces less than 3% of total AC activity in heart but eliminates Yotiao-associated AC activity. AC9^−/−^ mice exhibit no structural abnormalities but show a significant bradycardia, consistent with AC9 expression in sinoatrial node. Global changes in PKA phosphorylation patterns are not altered in AC9^−/−^ heart, however, basal phosphorylation of heat shock protein 20 (Hsp20) is significantly decreased. Hsp20 binds AC9 in a Yotiao-independent manner and deletion of AC9 decreases Hsp20-associated AC activity in heart. In addition, expression of catalytically inactive AC9 in neonatal cardiomyocytes decreases isoproterenol-stimulated Hsp20 phosphorylation, consistent with an AC9-Hsp20 complex. Phosphorylation of Hsp20 occurs largely in ventricles and is vital for the cardioprotective effects of Hsp20. Decreased Hsp20 phosphorylation suggests a potential baseline ventricular defect for AC9^−/−^. Doppler echocardiography of AC9^−/−^ displays a decrease in the early ventricular filling velocity and ventricular filling ratio (E/A), indicative of grade 1 diastolic dysfunction and emphasizing the importance of local cAMP production in the context of macromolecular complexes.

## Introduction

The second messenger cAMP and its effector proteins regulate numerous physiological processes in heart, including pacemaker activity, stress responses and cardiac contractility^1^. The majority of cAMP synthesis in heart, particularly in cardiac myocytes, is ascribed to two major adenylyl cyclase isoforms, AC5 and AC6. In mice, AC5 is important for parasympathetic regulation of cAMP production and cardiac stress responses, while AC6 appears to regulate aspects of calcium handling and cardiac contractility^1^. Deletion of both AC5/6 suggests these two enzymes control all beta-adrenergic enhancements of L-type calcium currents^2^. However, physiological roles for additional AC isoforms expressed at lower levels in heart are unknown.

We previously showed that AC9, an understudied largely forskolin-insensitive AC isoform, is expressed in adult mouse cardiomyocytes and forms complexes in heart with Yotiao, an A-kinase anchoring protein (AKAP)^3, 4^. AKAPs are important scaffolds that direct the localization, regulation, and integration of cAMP-dependent PKA signaling with downstream targets. Dysregulation of AKAP organized complexes can lead to cardiac remodeling and development of heart failure^5, 6^. For example, mAKAP (AKAP6) scaffolds AC5 to regulate cardiac stress responses while AKAP79 (AKAP5) scaffolds AC5/6 and L-type calcium channels^5, 7^. Association of AC with AKAP complexes serves to sensitize bound PKA substrates to the effects of cAMP, by up to two orders of magnitude^4, 8^.

In heart, AC9 is the only AC isoform to associate with Yotiao and the Yotiao-I_Ks_ channel complex^4^. The I_Ks_ channel results from the co-assembly of two subunits KCNQ1 and KCNE1. PKA phosphorylation of the anchored KCNQ1 channel subunit increases I_Ks_ current and shortens the action potential duration to allow sufficient diastolic intervals upon increased heart rate. Mutations in either KCNQ1 or Yotiao that disrupt their interaction give rise to Long-QT syndrome (LQT1, LQT11; a potentially lethal heritable arrhythmia syndrome)^9^. AC9 association with Yotiao-KCNQ1 facilitates KCNQ1 phosphorylation by PKA^4^. In humans, we suggest that AC9 is important for repolarization of heart. However since a functional I_Ks_ is largely absent in adult mice, additional potential roles for AC9 in heart are unknown.

In this report we show that AC9 accounts for less than 3% of total AC activity in mouse heart, yet represents all the Yotiao-associated AC activity. Loss of AC9 expression does not alter cardiac structure nor global PKA phosphorylation, but results in decreased PKA phosphorylation of heat shock protein 20 (Hsp20). PKA phosphorylated Hsp20 has previously been shown to be cardioprotective^10–12^. Molecular analyses indicate that Hsp20 is associated with AC9 in a Yotiao-independent manner. Loss of AC9 decreases Hsp20-associated AC activity in heart, while overexpression of catalytically inactive AC9 in neonatal cardiomyocytes decreases isoproterenol-stimulated Hsp20 phosphorylation. Finally, AC9 deletion gives rise to a grade 1a left ventricular diastolic dysfunction with preserved ejection fraction, consistent with a cardioprotective role for AC9.

## Results

### Genetic ablation of AC9 results in preweaning subviability

**Figure 1:**
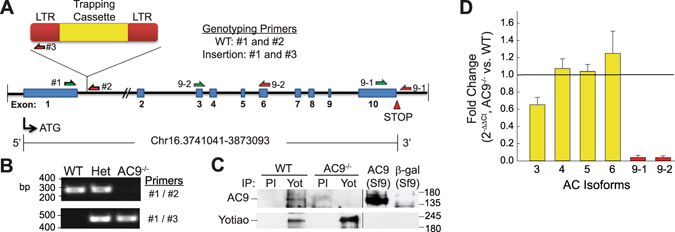
Design and verification of the AC9^−/−^ mouse model. (**A**) Schematic of AC9 gene-trap strategy and genotyping primers. Intron distances are not drawn to scale. (**B**) PCR analysis of genotyping: lanes 1, 2, and 3 represent wild-type (WT), AC9^+/−^ (Het) and AC9^−/−^, respectively. (**C**) AC9 protein levels were detected by immunoprecipitation of pre-immune (PI) or Yotiao complexes from WT and AC9 KO heart extracts followed by western blotting (WB) with anti-AC9 antibody. AC9 protein is not detectable in total heart extracts by WB (n = 5). (**D**) Real time PCR of cardiac AC isoforms in AC9^−/−^ heart, normalized to WT expression levels (n = 3; mice 1 month of age). Loss of AC9 mRNA was confirmed with primer sets 9-1 and 9-2. Full-length WBs are presented in Supplementary Fig. S6.

AC9 is ubiquitously expressed but physiological roles for AC9 have been largely ignored. To investigate the *in vivo* function of AC9, we utilized a gene-trap strain of AC9 obtained from the Mutant Mouse Regional Resource Center, a NIH strain repository. The AC9^−/−^ strain was created by Lexicon, Inc. using a retroviral insertion between exon 1 and 2 (Fig. 1A). The mouse genotypes were determined by PCR assay (Fig. 1B). AC9 protein is not detectable by western blotting in heart tissue homogenates and available antibodies against AC9 do not work well for immunoprecipitation. Therefore to confirm the lack of AC9 protein expression, we probed the Yotiao-AC9 complex which is tightly associated in mouse and guinea pig heart^4^. AC9 protein is detectable in immunoprecipitates of Yotiao from wild type (WT) hearts but not AC9^−/−^ (Fig. 1C). Multiple isoforms of AC are expressed in mouse adult cardiomyocytes, including AC 3, 4, 5, 6, and 9^4^; quantitative PCR of these AC isoforms from WT and AC9^−/−^ shows a 35 +/− 9% decrease in AC3 mRNA and complete loss of AC9, but no significant difference in other AC isoforms (Fig. 1D). AC3 protein was undetectable in lysates by western blotting.

Unpublished observations of FA Antoni suggested that conventional targeted deletion of AC9 results in early embryonic lethality in mice^13^. Although viable, we noted abnormal genotype frequencies for heterozygous mating pairs after backcrossing to C57BL/6 J (18% WT, 75% Het, 7% KO; n = 68, P = 0.002). The preweaning subviable homozygous phenotype with incomplete penetrance is also reported for another Adcy9^−/−^ strain (Adcy9tm1b(EUCOMM)Wtsi) created as part of the International Knockout Mouse Consortium^14^.

### Deletion of AC9 results in loss of Yotiao-associated AC but not significant changes in total AC activity

**Figure 2:**
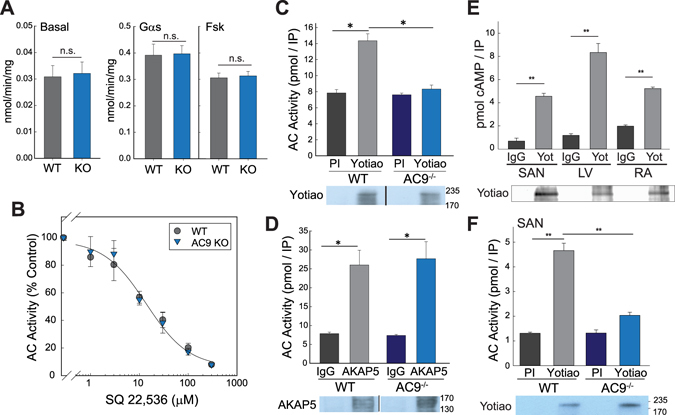
AC9 activity and AKAP association in heart. **(A**,**B**) Membranes were prepared from 6 wk WT versus AC9^−/−^ heart. (**A**) AC activity was measured under basal conditions and upon stimulation with 300 nM Gαs or 50 µM forskolin (n = 4, performed in duplicate or triplicate). (**B**) AC activity was measured in the presence of increasing concentrations of SQ22,536 in the presence of 300 nM Gαs (n = 3, performed in duplicate). (**C**,**D**) Heart extracts from WT or AC9^−/−^ mice were subjected to immunoprecipitation (IP) with pre-immune (control) or anti-Yotiao **(C)** and control IgG or anti-AKAP5 **(D)**. AKAP-associated AC activity was stimulated with 300 nM Gαs and measured (n = 3–4). Data are shown as mean +/− SD. A portion of the IP’s from (**C** and **D**) were subjected to WB analysis for the appropriate AKAP. (**E**) Yotiao-associated AC activity was measured from WT sinoatrial node (SAN), left ventricle (LV), and right atrium (RA) tissue homogenates as in panel (C). Immunoblot of Yotiao from IPs is shown below. (**F**) Yotiao-associated activity from WT and AC9^−/−^ SAN as measured in panel (C); Yotiao immunoblot is shown below (n = 3). Full-length WBs are presented in Supplementary Fig. S6.

AC9 mRNA and/or protein has previously been detected in both cardiac fibroblasts and myocytes^4, 15^, however the degree to which AC9 contributes to total AC activity is unknown. Initial measurements of basal and Gαs-stimulated AC activity showed no difference between cardiac membranes isolated from WT versus AC9^−/−^ mice (Fig. 2A). To potentially unmask AC activity stemming from AC9, we used a P-site inhibitor that displays >100 fold selectivity for AC5/6 over AC9 (Fig. 2B)^16^. No significant difference in total AC activity is observed, even at SQ 22,536 concentrations that inhibit 70–90% of AC5/6, but only 20–30% of AC9 (30–100 µM). From this data, we estimate that AC9 represents less than 3% of total heart AC activity. In order to detect AC9 activity, we examined its association with specific AKAP complexes^3, 4^. Heart extracts subjected to immunoprecipitation of Yotiao show significant AC activity that is pulled down with Yotiao in WT but not AC9^−/−^ (Fig. 2C). This is consistent with our previous findings that AC9 is the only AC isoform associated with Yotiao in heart^4^. Although AC9 can also bind AKAP79/150^17^, it does not significantly contribute to the AC activity associated with AKAP79/150 in heart (Fig. 2D).

### Reduced heart rate in the absence of AC9

Functional analysis of WT and AC9^−/−^ mice revealed a significant reduction of heart rate under isoflurane in both male and female mice for the two age groups examined (Table 1). Body weight is unaltered in male (21.1 +/− 0.2 versus 21.6 +/− 0.3 g, 6 months) and female mice (15.3 +/− 0.2 versus 15.6 +/− 0.2 g, 4 months). No structural abnormalities were noted and myocardial performance index (0.9 +/− 0.1 versus 0.9 +/− 0.1), ejection fraction (53 +/− 3 versus 51 +/− 4) and percent fractional shortening (27 +/− 2 versus 26 +/− 3) were all unchanged, as assessed by M mode imaging of mice 3–7 months (Supplemental Table 1). Alterations in heart rate suggest a role for AC9 in sinoatrial node (SAN). Yotiao-AC9 complex is detected throughout the heart, including the left ventricle, atria, and the SAN, as measured by AC9 activity associated with Yotiao immunoprecipitation (Fig. 2E). As shown for total heart, deletion of AC9 abolishes Yotiao-associated activity in SAN (Fig. 2F), consistent with a role for AC9 in heart rate control. Correct dissection of SAN is confirmed by the presence of connexin 45 but not connexin 43 (Supplemental Fig. S1)^18^.

**Table 1:** Deletion of AC9 gives rise to bradycardia.

Sex	Age (mo)	Average Heart Rate (bpm)		p-value
		WT	AC9^−/−^	
Male/Female	1–2	433 +/− 5 (n = 13/6)	409 +/− 7 (n = 12/6)	0.008
Male	5–7	445 +/− 6 (n = 10)	400 +/− 7 (n = 11)	0.0002

### Global PKA phosphorylation is unaltered but Hsp20 phosphorylation is decreased in AC9^−/−^

**Figure 3:**
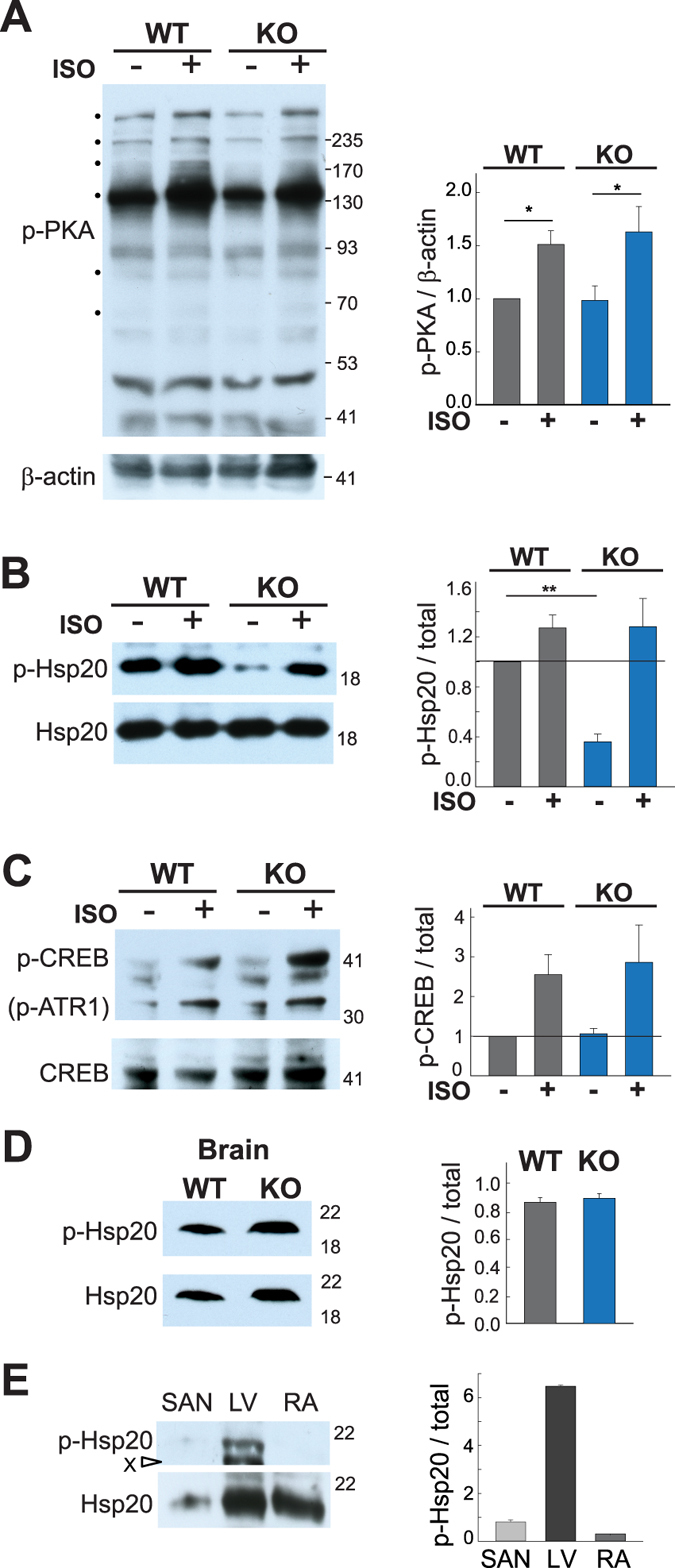
Decreased PKA phosphorylation of Hsp20 in AC9^−/−^. WT and AC9^−/−^ mice were injected with saline or isoproterenol (2 µg/g body weight, IP). Animals were sacrificed 4 min later and heart tissue was harvested. Cardiac extracts were prepared in the presence of phosphatase inhibitors. Equal protein supernatants were subjected to WB analysis with (**A**) anti-p-PKA substrate, (**B**) anti-p-Hsp20, and **C)** anti-p-CREB. Quantitation of phospho-PKA was normalized to beta-actin levels (A) while the corresponding total protein was quantitated by WB (n = 5–7) and the ratio of phosphoprotein to total was quantitated for p-Hsp20 (B) and p-CREB (**C**). (**D**,**E**) WB and quantitation of the ratio of phosphorylated to total Hsp20 in **D**) brain from WT and AC9^−/−^ mice (n = 3) and (**E**) sinoatrial node, left ventricle, and right atrium (n = 3). Graphs for quantitation of the ratio of phosphorylated to non-phosphorylated protein are shown to the right for each panel. **P < 0.01 t-test on raw intensity values. Full-length WBs are presented in Supplementary Fig. S6.

In order to determine if AC9 deletion alters cAMP signaling, we used intraperitoneal injection of saline or isoproterenol in WT and AC9^−/−^ mice to evaluate changes in phosphorylation of PKA targets. Global changes in PKA phosphorylation at baseline or after isoproterenol injection are not detected (Fig. 3A), suggesting that AC9 does not significantly alter sympathetic responses. However, we detect a large decrease in the basal phosphorylation state of Hsp20 in the absence of beta-adrenergic stimulation (Fig. 3B). Hsp20 is a known PKA target^19, 20^ and inhibition of PKA activity by H89 blocks isoproterenol-stimulated phosphorylation of Hsp20 in rat neonatal cardiomyocytes (Supplemental Fig. S2). Phosphorylation of other PKA targets such as troponin I, CREB, and phospholamban (PLN) are unaltered in the knockout (Fig. 3C, Supplemental Fig. S3). No change in basal Hsp20 phosphorylation was observed in WT and AC9^−/−^ brain lysates (Fig. 3D), suggesting that AC9 regulates Hsp20 in a tissue specific manner. Hsp20 total protein is present at very low levels in SAN node (only 11 +/− 9% of that in LV; Fig. 3E), while basal phosphorylation of Hsp20 is only significantly detected in left ventricle and not atrium (Fig. 3E).

**Figure 4:**
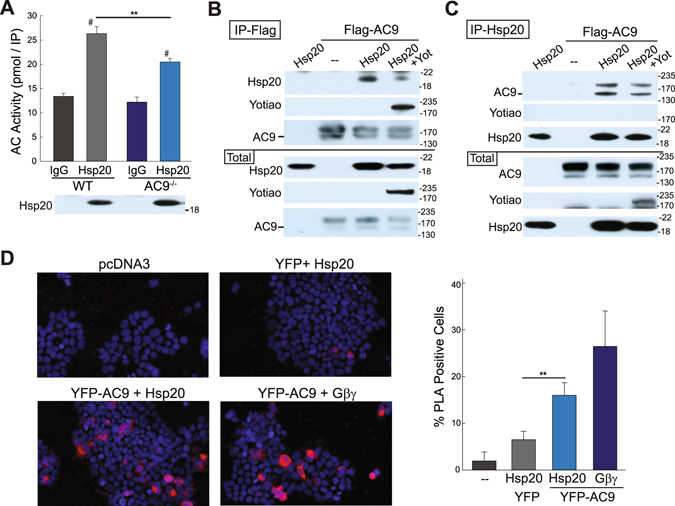
AC9 association with Hsp20 is Yotiao independent. (**A**) Heart extracts from WT or AC9^−/−^ mice were subjected to IP with IgG (control) or anti-Hsp20. Hsp20-associated AC activity was stimulated with 300 nM Gαs (n = 4; ^#^P < 0.001 IgG versus Hsp20; **P < 0.01 WT versus AC9^−/−^). (**B**,**C**) HEK293 cells were transfected with Flag-tagged AC9, myc-tagged Yotiao, and V5-tagged Hsp20 as indicated. Cell lysates were immunoprecipitated with anti-Flag (**B**) or anti-Hsp20 **(C**). AC9-Hsp20 protein complexes were detected by WB (n = 3). (**D**) Proximity ligation assay for Hsp20 and YFP-tagged AC9. HEK293 cells were transfected with the indicated plasmids. Representative images are shown. PLA signals were quantified by high content microscopy (positive cells defined as 4 signals or “dots” per cell; ~2000 cells imaged per condition). *P < 0.05 t-test, n = 3. Full-length WBs are presented in Supplementary Fig. S6.

To determine if AC9 is present in Hsp20-containing complexes, we measured the AC activity present in Hsp20 immunoprecipitates in WT and AC9^−/−^ heart extracts. Not only is a significant amount of AC activity complexed with Hsp20 in WT heart, this activity is decreased by 35 +/− 6% in AC9^−/−^ (Fig. 4A). Additional AC isoforms may associate with Hsp20 to facilitate isoproterenol-stimulated PKA phosphorylation. Interaction of AC9 and Hsp20 appears independent of Yotiao, as no detectable Hsp20 is associated with Yotiao in heart (Supplemental Fig. S4). AC9 and Hsp20 interactions can also be observed in HEK293 cells. A complex of AC9-Hsp20 is detected upon immunoprecipitation of either Flag-tagged AC9 or Hsp20 (Fig. 4B,C). Note, we consistently observe a 72 +/− 7% reduction of AC9-Hsp20 binding in the presence of Yotiao, suggesting that Yotiao may compete with Hsp20 for AC9 interactions. Finally, AC9-Hsp20 interactions are also detected by proximity ligation assay (PLA) in HEK293 cells (Fig. 4D). The high selectivity of PLA relies on double recognition of a protein complex by two oligonucleotide-conjugated secondary antibodies. Oligonucleotides in close proximity allows for rolling circle amplification that can be visualized (Fig. 4D, red dots). YFP-AC9 and Hsp20 display a significant signal by PLA as compared to YFP alone. Gβγ binds to the N-terminus of AC9 and serves as a positive control^21^.

**Figure 5:**
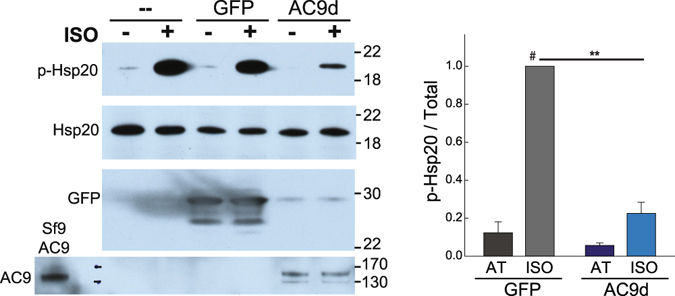
Expression of catalytically inactive AC9 decreases isoproterenol-stimulated phosphorylation of Hsp20 in rat neonatal cardiac myocytes (RNCMs). Cells were infected with GFP control or catalytically inactive AC9-D399A (AC9d) adenoviruses for 50 hr. RNCMs were treated with vehicle (AT) or isoproterenol (1 μM) for 5 min prior to cell lysis. The ratio of p-Hsp20 to total Hsp20 was quantitated by WB. ^#^, **P < 0.01 t-test, n = 4. Full-length WBs are presented in Supplementary Fig. S6.

AC9 binding to an Hsp20 complex should regulate local cAMP production and subsequent Hsp20 phosphorylation. If this is the case, displacing AC9 with a catalytically inactive enzyme would lead to reduced local cAMP and Hsp20 phosphorylation. Mutation of D399 to alanine deletes a key metal-binding residue in the active site of AC9, reducing Gαs-stimulated activity by >90% (Supplemental Fig. S5). Adenoviral expression of AC9-D399A in rat neonatal cardiomyocytes significantly decreased isoproterenol-stimulation of Hsp20 (by 77 +/− 6%), as compared to non-infected or GFP-infected cells (Fig. 5). This is consistent with decreased Hsp20 phosphorylation and Hsp20-associated AC activity in AC9^−/−^ heart.

### Protective role for AC9 against diastolic dysfunction

**Figure 6:**
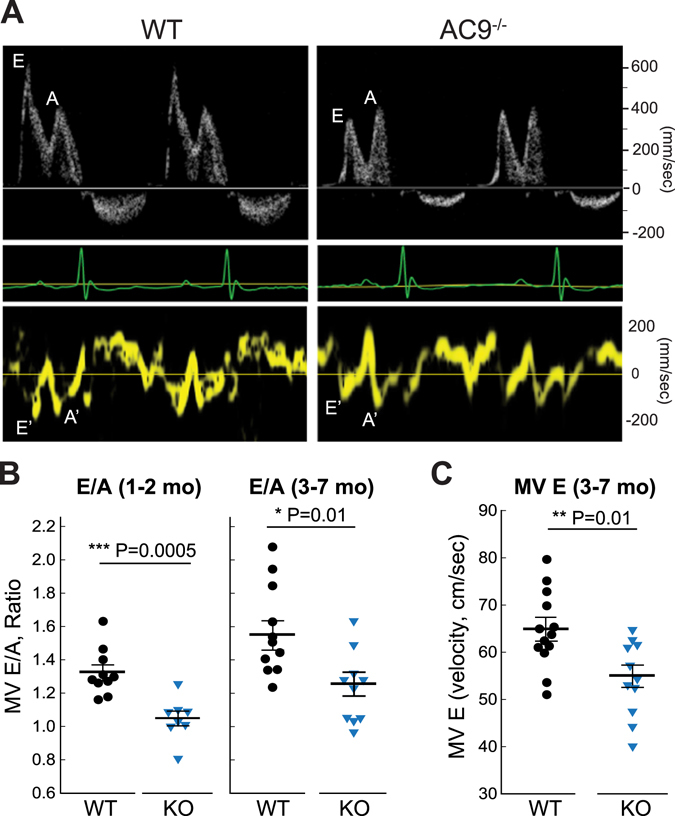
Cardiac parameters for WT and AC9^−/−^ mice. (**A**) Pulsed-wave and tissue Doppler recordings for female WT and AC9^−/−^ littermates (3 month) showing the relative amplitudes of the early ventricular filling (E wave) and the late filling caused by atrial contraction (A wave) (ECGs are shown below for each animal). (**B**) Quantitation of E/A ratio at 1–2 (n = 10 WT and 8 AC9^−/−^; males, P = 0.0005) or 3–7 months (n = 11 WT and 11 AC9^−/−^; males and females, P = 0.005). (**C**) Quantitation of mitral valve E wave at 3–7 months (n = 13 WT and 11 AC9^−/−^, P = 0.005). Mean +/− SE.

The decrease in baseline Hsp20 phosphorylation in AC9^−/−^ suggests a potential loss of the cardioprotective effects of PKA phosphorylated Hsp20^10–12^. Therefore, we measured overall left ventricular function using pulsed-wave Doppler echocardiography of early (E) and late (A) blood flow velocities through the mitral valve combined with tissue Doppler imaging of the mitral valve annulus (E’ and A’ velocity). Diastolic relaxation consists of four phases: isovolumetric relaxation, early rapid ventricular filling, slow filling or diastasis, and atrial contraction. Of these, the early filling phase (E wave) appears significantly reduced in AC9^−/−^ mice (Table 2 and Fig. 6). This was confirmed by tissue Doppler, measuring the early diastolic mitral annular velocity (E’) which is preload independent. Trends towards increased filling pressures (E/e’) are observed but never reach significance.

**Table 2:** Cardiac parameters for WT and AC9^−/−^ mice.

Parameter^#^	WT	AC9^−/−^	P value
**Mitral flow doppler**			
Peak velocity, E’ (mm/s)	20 +/− 1	15 +/− 1	0.004
Peak velocity, A’ (mm/s)	16.5 +/− 0.6	16.3 +/− 0.5	n.s.
Aortic ejection time (AET, ms)	51 +/− 2	52 +/− 1	n.s.
Isovolumic Relaxation Time (ms)	24 +/− 2	27 +/− 2	n.s.
Isovolumic Contraction Time (ms)	20 +/− 1	20 +/− 1	n.s.
Mitral valve A (cm/s)	41 +/− 2	43 +/− 2	n.s.
Mitral valve E (cm/s)	64 +/− 2	54 +/− 2	0.005
A’/E’	0.88 +/− 0.05	1.2 +/− 0.1	0.04
MV E/A	1.57 +/− 0.08	1.27 +/− 0.07	0.01
MV E/E’	33 +/− 1	38 +/− 3	n.s., 0.1
Myocardial Performance Index (MPI)	0.9 +/− 0.1	0.9 +/− 0.1	n.s.

^#^Mean +/− SE is given for male animals 3–7 months.

## Discussion

AC9 is the most divergent in sequence of the nine mammalian transmembrane AC isoforms. It is relatively insensitive to forskolin activation and remains the least characterized. It was originally cloned as a calcineurin-inhibitable AC isoform^22^, although it is unclear if calcineurin and other reported cellular regulators have direct or indirect mechanisms of action^23^. AC9 mRNA is ubiquitously expressed, particularly in the hippocampus^24^, and the knockout of AC9 was thought to be embryonic lethal^13^. Therefore, physiological roles for AC9 have been largely ignored. However, despite a preweaning subviability, AC9^−/−^ mice show no obvious size or structural abnormalities. AC9 expression is important for human neutrophil chemotaxis^25^, while ADCY9 gene polymorphisms are linked to asthma, mood disorders, and body weight^26–28^. In heart, AC9 mRNA and/or protein has been detected in both cardiomyocytes and fibroblasts, however, our estimates using P-site inhibitors suggest that AC9 represents less than 3% of total heart AC activity. Despite the low levels of AC9, Yotiao-associated AC activity is abolished in the knockout while AKAP79-associated AC activity is unchanged. Although AKAP79 can bind to AC9 in cell culture experiments, AKAP79 scaffolds the highly expressed AC5/6 isoforms in heart^17, 29^. The association with Yotiao is consistent with a role for AC9 in regulation of I_Ks_ channels and potentially long QT syndrome, for which mutations of I_Ks_ and Yotiao are well known^9^.

### Bradycardia

LQT1 patients (KCNQ1 mutations) often display reduced heart rate, particularly with exercise^30, 31^. AC9 is proposed to regulate PKA phosphorylation of KCNQ1 via its scaffolding to Yotiao^4^, however, the interpretation of a bradycardia phenotype is complicated in mice. Unlike humans, standard I_Ks_ currents are largely absent in adult mouse myocytes^32–34^. We do not detect endogenous KCNQ1 from heart lysates or by immunoprecipitation of Yotiao or KCNQ1^4^. Moreover, knockout of KCNQ1 does not alter heart rate or QT interval at baseline, although these features are prolonged when challenged with nicotine^35^. Thus it is unclear if the bradycardia in AC9^−/−^ is due to decreased phosphorylation of KCNQ1 or another, yet identified, Yotiao-associated K^+^ channel in mouse sinoatrial node.

### AC9 binds Hsp20 and regulates basal Hsp20 phosphorylation

Although global PKA phosphorylation is unchanged, deletion of AC9 has a pronounced effect on the basal phosphorylation of Hsp20. Despite its name, Hsp20 (also known as HspB6) is not heat inducible but belongs to a group of ubiquitously expressed small heat shock proteins that are abundant in cardiac, smooth, and skeletal muscle^36, 37^. Hsp20 is up-regulated in response to cellular stress and/or damage and its expression is cardioprotective against prolonged beta-agonist induced hypertrophy, ischemia/reperfusion injury, and the cardiotoxic effects of doxorubicin^36–38^. Phosphorylation of Ser16 on Hsp20 by PKA is increased under cardiac stress and is essential for its protective effects after ischemia/reperfusion injury^10–12, 39^. Hsp20 is found in association with numerous complexes in heart including actin/α-actinin, 14-3-3, phosphorylated Akt, PP1-PLN, and members of an AKAP-Lbc complex implicated in the development of cardiac hypertrophy (reviewed in refs 36, 37 and 40). Disruption of Hsp20 association with PDE4D^39^, PKD1^41^, or AKAP-Lbc^42^ abolish the protective effects of Hsp20 in the heart.

Hsp20 is associated with AC9 in heart and in HEK293 cells, as determined by IP and PLA assays. It is not clear if AC9 directly binds Hsp20 or if the association is mediated by an Hsp20 binding partner. However, we see no evidence for Hsp20 interaction with Yotiao in heart; in HEK293 cells, expression of Yotiao actually reduces AC9-Hsp20 interactions. Although AC9 clearly binds Hsp20, other AC isoforms in heart must also associate with Hsp20-containing complexes, as 65 +/− 4% of Hsp20-associated AC activity still remains in AC9^−/−^ heart. Expression of a catalytically inactive mutant of AC9 in neonatal cardiomyocytes decreases isoproterenol-stimulated Hsp20 phosphorylation by 77 +/− 6%. Therefore, overexpression of catalytically inactive AC9 may prevent Hsp20 from associating with endogenous AC9 and other AC isoforms in cardiomyocytes, reducing local cAMP production necessary for PKA phosphorylation of Hsp20.

### Protective role for AC9 against diastolic dysfunction

Despite the fact that AC9 associates with the Yotiao-I_Ks_ complex, it wasn’t clear if deletion of AC9 would display alterations in the relaxation of heart muscle in mice, given the reported lack of I_Ks_ currents. Surprisingly, the early filling phase (E wave) of diastolic relaxation appears significantly reduced in AC9^−/−^ mice. This was confirmed by tissue Doppler imaging. A slower E wave is associated with a stiffer left ventricular wall, increasing the back pressure to slow blood flow velocity. This gives rise to a lower E/A ratio in AC9^−/−^, an accepted clinical marker of diastolic dysfunction. The resulting phenotype is categorized as a grade 1a with preserved ejection fraction. Patients with LQTS (LQT1 and LQT2) also show diastolic dysfunction, with a significantly slower E’ and increased left atrial volume index as compared to healthy subjects^43^. However, the QT interval is not increased in AC9^−/−^, suggesting the lower E/A ratio may not reflect altered K^+^ channel regulation involved in repolarization, but rather the loss of Hsp20 phosphorylation and its cardioprotective effects.

## Conclusion

Despite contributing to very low overall cAMP production in heart, loss of AC9 reduces Yotiao- and Hsp20-localized pools of AC activity, but not global PKA phosphorylation. Phosphorylation of Hsp20 occurs largely in ventricles and is vital for the cardioprotective effects of Hsp20^36–38^. Deletion of AC9 results in a left ventricular diastolic dysfunction which correlates with reduced Hsp20-associated AC activity and a significant 64% reduction in baseline Hsp20 phosphorylation. Reductions in AC9 mRNA and protein also occur via microRNA miR-142-3p^44, 45^. Although normally low in heart, miR-142-3p is upregulated in patients with non-ischemic dilated cardiomyopathy, and in mouse models of hypertrophic cardiomyopathy^46–49^, consistent with a protective role for AC9 in heart.

## Materials and Methods

### Generation of AC9 Gene-Targeted Mice

The mouse strain used for this research project, B6;129S5-*Adcy9Gt*(*neo*)*159Lex*/Mmucd, identification number 011682-UCD, was obtained from the Mutant Mouse Regional Resource Center, a NIH funded strain repository, and was donated to the MMRRC by Lexicon, Inc. The insertion of the gene trap vector was generated in strain 129/SvEvBrd-derived embryonic stem cells^14^. The retroviral insertion (5174bp) occurred in the intron between exons 1 and 2. The chimeric mice were bred to C57BL/6J mice to generate F1 heterozygous animals. Mice were backcrossed with C57BL/6J mice for 7–9 generations. Age matched or wild-type C57BL/6J littermate controls were used as described. All animal protocols were approved by the Institutional Animal Care and Use Committee (IACUC) at the University of Texas Health Science Center at Houston in accordance with the Animal Welfare Act and NIH guidelines.

### Genotyping and RT-PCR

Primers used to detect gene trap insertion (WT #1 and KO #3; 494 bp) or WT animals (WT #1 and #2; 280 bp) were as follows: KO #3, GGCCAAGAACAGATGGAACAG; WT #1, TCCCTAGCCATTCCTAGCAAAGC; WT #2, CAGTTCACCTTTTCCATACCCCTAG. Primers used for real-time PCR are from^50^, except AC9. Primer sequences for AC9 are as follows (see Fig. 1): AC9-1 Fwd CGGTCTCCCACAGATGAGAT; AC9-1 Rev, TCTGGGGACAGAAACTGAGG; AC9-2 Fwd, CTTTGATAACCTTAAGACTTGC; AC9-2 Rev, CAGGAGCTGGAGCGATCATA. Real-time PCR was performed and analyzed as described^51^, using GAPDH as a control template.

### Plasmids and adenoviruses

Myc-Yotiao-pcDNA3 was previously described^3^. Myc-tagged Hsp20 was purchased from Origene; V5-Hsp20 was a gift from Dr. George Baillie (U of Glasgow). A flag-tag (MDYKDDDDK) plus two residue linker (GA) was inserted in frame at the N-terminus of human AC9 using nested PCR primers. The resulting clone was sequenced and the activity of the tagged protein verified upon expression in HEK293 cells and Sf9 cells. YFP-tagged AC9 was similarly created using flag-AC9 pCDNA3 as the starting construct and replacing the flag-tag with YFP. To create a catalytically inactive AC9, aspartate 399 was mutated to alanine using QuikChange II Site-Directed Mutagenesis Kit (Agilent Technologies). For adenoviral expression, GFP and YFP-tagged catalytically inactive (AC9-D399A, AC9d) were inserted into the Kpn I/XbaI restriction sites of pShuttle-CMV vector. Recombinant adenoviruses were produced according to the manufacturer’s instructions (AdEasy Adenoviral Vector Systems, Stratagene). Appropriate clones were selected by RT-PCR and sequenced. Note, although AC9d is expressed as YFP-tagged, YFP is typically cleaved when expressed by adenoviruses in cardiomyocytes and the YFP tag is not detected by WB.

### Cell culture and transfections

HEK293 cells were authenticated by ATCC, cultured in Dulbecco’s Modified Eagle Medium with 10% fetal bovine serum, and transfected with the indicated plasmids using Lipofectamine 2000^3, 17^. Neonatal rat ventricular myocytes (NRVM) were isolated from 1- to 2-day-old Sprague-Dawley rat hearts as previously described^52^. Medium was changed 24 hours after plating and 48–72 hrs post isolation NRVMs were infected with adenovirus (multiplicity of infection of 50–100) for 50 hrs prior to treatments. Experiments were carried out on at least three separate NRVM isolations. Isoproterenol was stored and diluted in AT buffer (100 mM ascorbate and 10 mM thiourea, pH 7.4).

### Western Blotting

Antibodies used for immunoprecipitation and western blotting include rabbit anti-Hsp20 (phospho S16, Abcam), mouse anti-Hsp20 (Hsp20-11) (Santa Cruz Biotechnology), rabbit anti-phospho-Troponin I (Cardiac) (Ser23/24, Cell Signaling), rabbit anti-Troponin I (Cell signaling), rabbit anti-phospho-Phospholamban (Ser16, EMD Millipore), mouse anti-Phospholamban Antibody (2D12, ThermoFisher Scientific), rabbit anti-phospho-PKA Substrate (RRXS*/T*) (100G7E) (Cell Signaling), mouse anti-β-actin (C4, Santa Cruz Biotechnology), rabbit anti- CREB (48H2, Cell Signaling), rabbit anti-phospho-CREB (Ser133, 87G3, Cell signaling), goat anti-cyclase IX (N-18, Santa Cruz Biotechnology), mouse or rabbit anti-AKAP150 (EMD Millipore for western blotting and Santa Cruz Biotechnology for immunoprecipitation) and normal mouse or rabbit IgG (Santa Cruz Biotechnology). Connexin 43 (rabbit) and connexin 45 (mouse) antibodies were a gift from Dr. John O’Brien (UTHealth). The rabbit anti-Yotiao antibody^3^ and mouse anti-AC5 antibodies^51^ were generated and characterized as described. Full-length western blots are shown in Supplemental Fig. S6.

For analysis of PKA phosphorylation in heart, WT and AC9^−/−^ mice were injected with saline or isoproterenol (2 µg/g body weight, IP). Animals were sacrificed 4 min later and heart tissue was harvested. Cardiac extracts were prepared in the presence of phosphatase inhibitors. Equal protein supernatants were subjected to western blot analysis as described in figure legends.

### Adenylyl Cyclase Activity and IP-AC Assays

Preparation of heart extracts and measurement of AC activity were performed as previously described^3, 17^. AC9 activity in WT hearts was estimated from increasing concentrations of the SQ22,536 inhibitor that displays >100 fold selectivity for AC5/6 over AC9^16^. At X concentration of SQ22,536, AC9 activity = (WT-KO)/%AC9 activity remaining at X concentration. Averages of 4 experiments, performed in duplicate or triplicate, using 10, 30, 100, and 300 μM concentrations of SQ22,536 were used for estimates; a 0.08–2.5% difference in activity is observed between WT and AC9KO heart membranes. Note, at zero SQ22,536 there is no detectable difference in activity. Immunoprecipitation of AKAP or Hsp20 complexes followed by western blotting or measurement of associated AC activity was performed as described^53^. AC activity was stimulated with the indicated reagents and cAMP was detected by enzyme immunoassay (Assay Designs) or using [γ^32^P]ATP.

### Proximity Ligation Assay

*In situ* PLA was performed using a Duolink kit (Sigma-Aldrich, cat. DUO92101) following the manufacturer’s protocol. HEK293 cells were cultured on clear bottom 96 well plates (Greiner Bio-One), transfected with the required plasmids and fixed with 4% PFA. After washing the plate 3 times with PBS, the cells were blocked (1% BSA + 0.075% Triton X100) for 1 h at room temperature and then incubated with primary antibodies overnight. Antibodies included: mouse anti-Hsp20 (SC-51955, SantaCruz, 1:500), mouse anti-GFP (632381, Living colors, 1:500), rabbit anti-GFP (SC-8334, SantaCruz, 1:1000), and mouse anti-Gβγ (SC-378, SantaCruz, 1:1000). After removal of primary antibodies, the samples were incubated with anti-mouse PLUS and anti-rabbit MINUS PLA probes for 1 h at 37 °C. Subsequent steps of ligation and amplification were according to the manufacturer’s protocol. After the last wash, cells were stained with DAPI (1 μg/ml) and imaged using an epifluorescence high content imaging microscope with a 20X objective (CellInsight CX5 High Content Screening platform, ThermoFisher). Data analysis was performed using FACS analysis software (FlowJo, USA). To prevent false positives, cells with saturating YFP fluorescence or less than 4 positive signals (dots) per cell were not considered in the analysis.

### Echocardiography

AC9 knockout and WT mice (3–7 months) were anesthetized in a chamber under 2.5% isoflurane. After sedation, mice were transferred to a heated platform and fixed in the prone position for electrocardiogram recordings and cardiac imaging. Anesthesia was administered via a nose cone at 1–1.5% isoflurane during recordings. Cardiac imaging was utilized to assess left ventricular (LV) diastolic function and other key cardiac parameters. Initial analysis of LV diastolic function was evaluated with pulsed-wave Doppler (PWD) (20 MHz, Doppler Signaling Processing Workstation (DSPW), Indus Instruments, Webster, USA) of the mitral valve (MV) inflow^54^. Key parameters of the mitral valve flow profile including, early (E) and late (A) filling velocities, aortic ejection time (AET), isovolumetric relaxation time (IVRT), and isovolumetric contraction time (IVCT) were determined from recordings with DSPW. The ratio of the E/A velocities was used to assess changes in LV filling.

Further evaluation of LV diastolic function and key cardiac function used high resolution ultrasound (40 MHz; Vevo 3100, Visual Sonics Inc., Toronto, Canada); analysis was performed in VevoLab (VisualSonic). LV volume and mass, ejection fraction (EF), and fractional shortening (FS) were calculated from recordings of the short axis using M-mode imaging. Additional measurements from M-mode imaging included the thickness of the LV anterior and posterior wall (LVAW and LVPW) during both systole and diastole. Diastolic function was assessed with both PWD and tissue Doppler imaging (TDI), visualized in apical four-chamber view. IVCT, IVRT, AET, E and A velocities, myocardial performance index (MPI), and E/A ratios were determined from PWD recordings. TDI from the septal side of the mitral annulus were used to assess the early (E’) and late (A’) velocities of the mitral annulus and the ratio of E’/A’, A’/E’, and MV E/E’. All measurements were averaged from 5 cardiac cycles for each parameter. Note, differences in MV E/A and E’/A’ ratios are likely underestimates since the A’ and MV A measurements were not always possible on WT mice with faster heart rates where the early wave is dominant; this was never an issue with AC9^−/−^ mice.

### Statistical Analysis

Data are expressed as mean ± standard error of the mean (SEM), except where noted. Differences between samples were determined using two-way analysis of variance (ANOVA) followed by Bonferroni’s Multiple Comparison Test for comparison between multiple groups, or unpaired t test for comparison between two groups. Chi square test was used to compare observed and expected genotype frequencies. Significant p values are indicated as follows: (*) denotes a p value < 0.05, (**) < 0.01 and (***) < 0.001. All analyses were performed using Excel or SigmaPlot statistical analysis software.

## Electronic supplementary material

Supplemental material and figures

## References

[1] Sadana R, Dessauer CW (2009). Physiological Roles for G Protein-Regulated Adenylyl Cyclase Isoforms: Insights from Knockout and Overexpression Studies. NeuroSignals.

[2] Timofeyev V (2013). Adenylyl cyclase subtype-specific compartmentalization: differential regulation of L-type Ca2+ current in ventricular myocytes. Circ Res.

[3] Piggott LA, Bauman AL, Scott JD, Dessauer CW (2008). The A-kinase anchoring protein Yotiao binds and regulates adenylyl cyclase in brain. Proc Natl Acad Sci USA.

[4] Li Y, Chen L, Kass RS, Dessauer CW (2012). The A-kinase anchoring protein Yotiao facilitates complex formation between type 9 adenylyl cyclase and the IKs potassium channel in heart. J Biol Chem.

[5] Efendiev R, Dessauer CW (2011). A kinase-anchoring proteins and adenylyl cyclase in cardiovascular physiology and pathology. J Cardiovasc Pharmacol.

[6] Scott JD, Dessauer CW, Tasken K (2013). Creating order from chaos: cellular regulation by kinase anchoring. Annu Rev Pharmacol Toxicol.

[7] Dessauer CW (2009). Adenylyl cyclase–A-kinase anchoring protein complexes: the next dimension in cAMP signaling. Mol Pharmacol.

[8] Efendiev R, Bavencoffe A, Hu H, Zhu MX, Dessauer CW (2013). Scaffolding by A-kinase anchoring protein enhances functional coupling between adenylyl cyclase and TRPV1 channel. J Biol Chem.

[9] Ackerman MJ, Mohler PJ (2010). Defining a new paradigm for human arrhythmia syndromes: phenotypic manifestations of gene mutations in ion channel- and transporter-associated proteins. Circ Res.

[10] Qian J (2009). Blockade of Hsp20 phosphorylation exacerbates cardiac ischemia/reperfusion injury by suppressed autophagy and increased cell death. Circ Res.

[11] Edwards HV, Scott JD, Baillie GS (2012). PKA phosphorylation of the small heat-shock protein Hsp20 enhances its cardioprotective effects. Biochem Soc Trans.

[12] Nicolaou P (2008). Human mutation in the anti-apoptotic heat shock protein 20 abrogates its cardioprotective effects. J Biol Chem.

[13] Antoni FA (2006). Adenylyl cyclase type 9. UCSD-Nature Molecule Pages.

[14] International Mouse Knockout C, Collins FS, Rossant J, Wurst W (2007). A mouse for all reasons. Cell.

[15] Ostrom RS (2003). Angiotensin II enhances adenylyl cyclase signaling via Ca2+/calmodulin. Gq-Gs cross-talk regulates collagen production in cardiac fibroblasts. J Biol Chem.

[16] Brand CS, Hocker HJ, Gorfe AA, Cavasotto CN, Dessauer CW (2013). Isoform selectivity of adenylyl cyclase inhibitors: characterization of known and novel compounds. J Pharmacol Exp Ther.

[17] Efendiev R (2010). AKAP79 interacts with multiple adenylyl cyclase (AC) isoforms and scaffolds AC5 and -6 to alpha-amino-3-hydroxyl-5-methyl-4-isoxazole-propionate (AMPA) receptors. J Biol Chem.

[18] Severs NJ (2004). Gap junction alterations in human cardiac disease. Cardiovasc Res.

[19] Fan GC, Chu G, Kranias EG (2005). Hsp20 and its cardioprotection. Trends Cardiovasc Med.

[20] Beall A (1999). The small heat shock-related protein, HSP20, is phosphorylated on serine 16 during cyclic nucleotide-dependent relaxation. J Biol Chem.

[21] Brand CS, Sadana R, Malik S, Smrcka AV, Dessauer CW (2015). Adenylyl Cyclase 5 Regulation by Gbetagamma Involves Isoform-Specific Use of Multiple Interaction Sites. Mol Pharmacol.

[22] Paterson JM, Smith SM, Harmar AJ, Antoni FA (1995). Control of a novel adenylyl cyclase by calcineurin. Biochem.Biophys.Res.Commun..

[23] Dessauer CW (2017). International Union of Basic and Clinical Pharmacology. CI. Structures and Small Molecule Modulators of Mammalian Adenylyl Cyclases. Pharmacol Rev.

[24] Antoni FA (1998). Ca2+/calcineurin-inhibited adenylyl cyclase, highly abundant in forebrain regions, is important for learning and memory. Journal of Neuroscience.

[25] Liu L, Das S, Losert W, Parent CA (2010). mTORC2 regulates neutrophil chemotaxis in a cAMP- and RhoA-dependent fashion. Dev Cell.

[26] Small KM (2003). An Ile to Met polymorphism in the catalytic domain of adenylyl cyclase type 9 confers reduced beta2-adrenergic receptor stimulation. Pharmacogenetics.

[27] Toyota T (2002). Molecular analysis, mutation screening, and association study of adenylate cyclase type 9 gene (ADCY9) in mood disorders. Am J Med Genet.

[28] Berndt SI (2013). Genome-wide meta-analysis identifies 11 new loci for anthropometric traits and provides insights into genetic architecture. Nature genetics.

[29] Nichols CB (2010). Sympathetic stimulation of adult cardiomyocytes requires association of AKAP5 with a subpopulation of L-type calcium channels. Circ Res.

[30] Swan H (1999). Sinus node function and ventricular repolarization during exercise stress test in long QT syndrome patients with KvLQT1 and HERG potassium channel defects. J Am Coll Cardiol.

[31] Schwartz PJ (2008). Neural control of heart rate is an arrhythmia risk modifier in long QT syndrome. J Am Coll Cardiol.

[32] Honore E (1991). Cloning, expression, pharmacology and regulation of a delayed rectifier K+ channel in mouse heart. EMBO J.

[33] Salama G, Baker L, Wolk R, Barhanin J, London B (2009). Arrhythmia phenotype in mouse models of human long QT. J Interv Card Electrophysiol.

[34] Marx SO (2002). Requirement of a macromolecular signaling complex for beta adrenergic receptor modulation of the KCNQ1-KCNE1 potassium channel. Science.

[35] Tosaka T (2003). Nicotine induces a long QT phenotype in Kcnq1-deficient mouse hearts. J Pharmacol Exp Ther.

[36] Fan GC, Kranias EG (2011). Small heat shock protein 20 (HspB6) in cardiac hypertrophy and failure. J Mol Cell Cardiol.

[37] Martin TP, Currie S, Baillie GS (2014). The cardioprotective role of small heat-shock protein 20. Biochem Soc Trans.

[38] Fan GC (2008). Heat shock protein 20 interacting with phosphorylated Akt reduces doxorubicin-triggered oxidative stress and cardiotoxicity. Circ Res.

[39] Martin TP (2014). Targeted disruption of the heat shock protein 20-phosphodiesterase 4D (PDE4D) interaction protects against pathological cardiac remodelling in a mouse model of hypertrophy. FEBS Open Bio.

[40] Dreiza CM (2010). The small heat shock protein, HSPB6, in muscle function and disease. Cell Stress Chaperones.

[41] Sin YY, Martin TP, Wills L, Currie S, Baillie GS (2015). Small heat shock protein 20 (Hsp20) facilitates nuclear import of protein kinase D 1 (PKD1) during cardiac hypertrophy. Cell Commun Signal.

[42] Edwards HV, Scott JD, Baillie GS (2012). The A-kinase-anchoring protein AKAP-Lbc facilitates cardioprotective PKA phosphorylation of Hsp20 on Ser(16). Biochem J.

[43] Leren IS (2015). Cardiac Mechanical Alterations and Genotype Specific Differences in Subjects With Long QT Syndrome. JACC: Cardiovascular Imaging.

[44] Huang B (2009). miR-142-3p restricts cAMP production in CD4+ CD25- T cells and CD4+ CD25+ TREG cells by targeting AC9 mRNA. EMBO Rep.

[45] Lv M (2012). An oncogenic role of miR-142-3p in human T-cell acute lymphoblastic leukemia (T-ALL) by targeting glucocorticoid receptor-alpha and cAMP/PKA pathways. Leukemia.

[46] Tijsen AJ, Pinto YM, Creemers EE (2012). Circulating microRNAs as diagnostic biomarkers for cardiovascular diseases. Am J Physiol Heart Circ Physiol.

[47] Voellenkle C (2010). MicroRNA signatures in peripheral blood mononuclear cells of chronic heart failure patients. Physiol Genomics.

[48] Bagnall RD, Tsoutsman T, Shephard RE, Ritchie W, Semsarian C (2012). Global MicroRNA Profiling of the Mouse Ventricles during Development of Severe Hypertrophic Cardiomyopathy and Heart Failure. PLoS One.

[49] Baskerville S, Bartel DP (2005). Microarray profiling of microRNAs reveals frequent coexpression with neighboring miRNAs and host genes. RNA.

[50] Landa LR (2005). Interplay of Ca2+ and cAMP signaling in the insulin-secreting MIN6 beta-cell line. J Biol Chem.

[51] Bavencoffe A (2016). Persistent Electrical Activity in Primary Nociceptors after Spinal Cord Injury Is Maintained by Scaffolded Adenylyl Cyclase and Protein Kinase A and Is Associated with Altered Adenylyl Cyclase Regulation. J Neurosci.

[52] Wu HC (2015). Identification and characterization of two ankyrin-B isoforms in mammalian heart. Cardiovascular Research.

[53] Li, Y. & Dessauer, C. W. In *Cyclic Nucleotide Signaling* (eds Cheng, X.) Ch. 9, 147–164 (CRC Press, 2015).

[54] Reddy AK (2005). Pulsed Doppler signal processing for use in mice: design and evaluation. IEEE Trans Biomed Eng.

