# Supplementary material for: Loss of type 9 adenylyl cyclase triggers reduced phosphorylation of Hsp20 and diastolic dysfunction
Source: Sci Rep. 2017 Jul 17;7:5522. doi: 10.1038/s41598-017-05816-w (PMC5514062; doi:10.1038/s41598-017-05816-w)

Loss of type 9 adenylyl cyclase triggers reduced phosphorylation of Hsp20 and diastolic dysfunction

Yong Li[1], Tanya A. Baldwin[1], Yan Wang, Janani Subramaniam[1], Anibal Garza Carbajal[1], Cameron S Brand[2], Shane R Cunha[1], and Carmen W Dessauer[1,*]

[1]Dept. Integrative Biology and Pharmacology, McGovern Medical School, University of Texas Health Science Center, Houston, TX 77030

Supplementary Table 1. Cardiac parameters for WT and AC9$^{-/-}$ mice.

Parameter[#]	WT	AC9$^{-/-}$	P value
**M-mode Echocardiography**			
LV anterior wall, diastole (mm)	0.70 +/- 0.06	0.72 +/- 0.05	n.s.
LV anterior wall, systole (mm)	0.90 +/- 0.08	1.01 +/- 0.08	n.s.
LV internal diastolic diameter (mm)	3.6 +/- 0.1	3.7 +/- 0.1	n.s.
LV internal systolic diameter (mm)	2.6 +/- 0.1	2.7 +/- 0.1	n.s.
LV posterior diastolic wall (mm)	0.76 +/- 0.08	0.69 +/- 0.04	n.s.
LV posterior systolic wall (mm)	0.94 +/- 0.06	1.14 +/- 0.07	n.s.
EF (%)	53 +/- 3	51 +/- 4	n.s.
FS (%)	27 +/- 2	26 +/- 3	n.s.
LV Mass, corrected (mg)	73 +/- 12	73 +/- 7	n.s.
End diastolic volume (µL)	55 +/- 4	58 +/- 4	n.s.
End systolic volume (µL)	26 +/- 3	28 +/- 3	n.s.

Supplementary Figure Legends.

Fig S1. SAN shows connexin 45 (Cx45) but not Cx43 expression.

Fig S2. Inhibition of PKA abolishes isoproterenol-stimulated phosphorylation of Hsp20. Rat neonatal cardiomyocytes were pretreated in the absence or presence of 10 μM of the PKA inhibitor H89 for 10 min followed by vehicle (AT) or isoproterenol (1 μM) for 5 min. Cells were lysed and subjected to WB analysis for phosphorylation of Hsp20 (n=3).

Fig S3. Deletion of AC9 does not alter PKA phosphorylation of Troponin I and phospholamban. WT and $AC9^{-/-}$ mice were injected with saline or isoproterenol (2 μg/g body weight, IP). Animals were sacrificed 4 min later and heart tissue was harvested. Cardiac extracts were prepared in the presence of phosphatase inhibitors. Equal protein supernatants were subjected to WB analysis with **A)** anti-p-Troponin 1 and **B)** anti-p-PLN. The corresponding total protein was quantitated by WB (n=5-7) and the ratio of phosphoprotein to total is shown for each replicate.

Fig S4. Immunoprecipitation of Yotiao fails to pull-down Hsp20 in heart. IP of heart extracts from WT or $AC9^{-/-}$ mice with rabbit IgG (control) or anti-Yotiao were subjected to WB analysis for Hsp20 and Yotiao (n=3).

Fig S5. Mutation of D399A in AC9 has dramatically reduced catalytic activity. Membranes were prepared from Sf9 cells expressing β-gal, AC9, or AC9-D399A. AC activity was measured upon stimulation with 300 nM GTPγS-Gαs (n=3). WB of membrane proteins is shown.

Fig S6. Full-length western blots for figures 1-5 in the main paper.

Supplementary Figures.

Fig S1

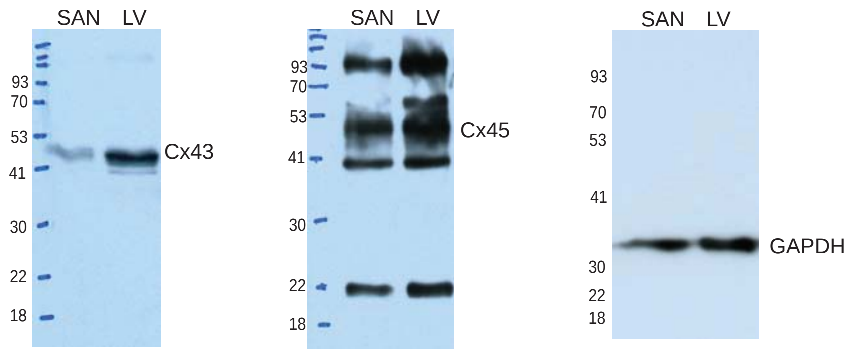

Fig S2

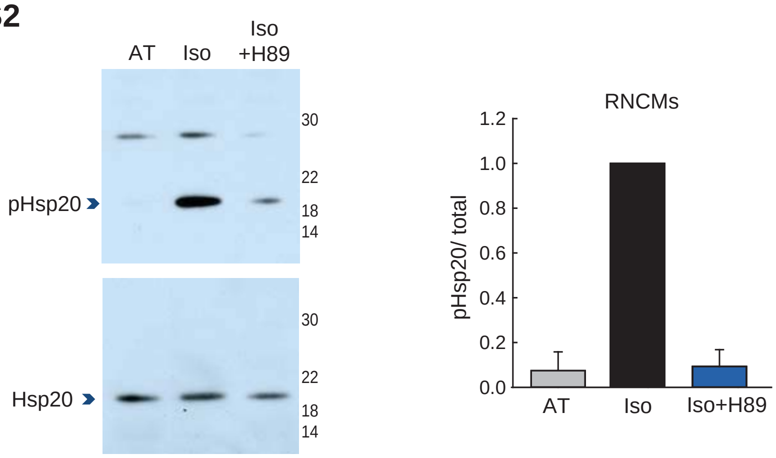

Fig S3

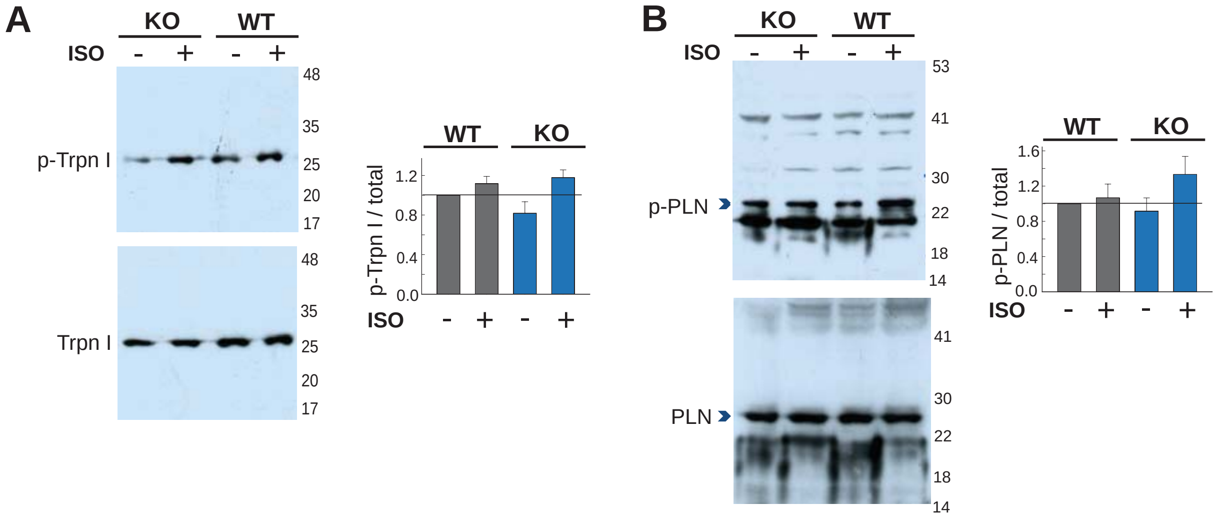

Fig S4

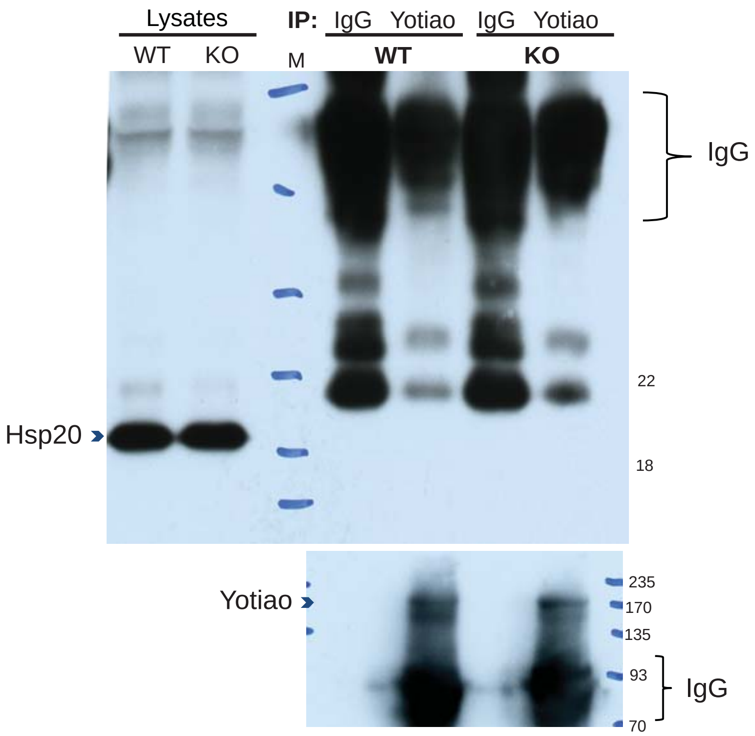

Lysates
IP: IgG Yotiao IgG Yotiao
WT KO
M
WT
KO
IgG
22
18
Hsp20
Yotiao
235
170
135
93
70
IgG

Fig S5

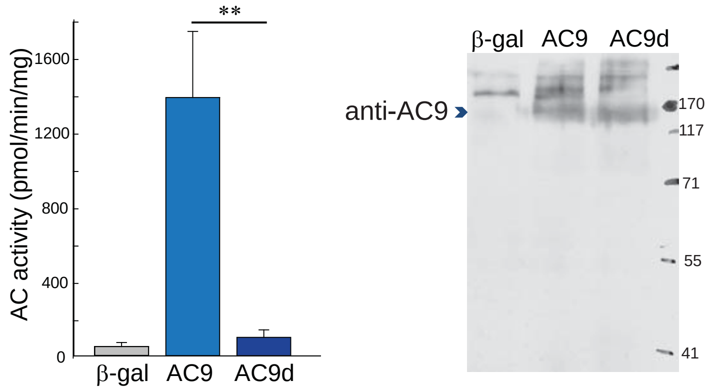

**
AC activity (pmol/min/mg)
1600
1200
800
400
0
β-gal AC9 AC9d
β-gal AC9 AC9d
anti-AC9
170
117
71
55
41

Supplemental Fig 6. Full length western blots.

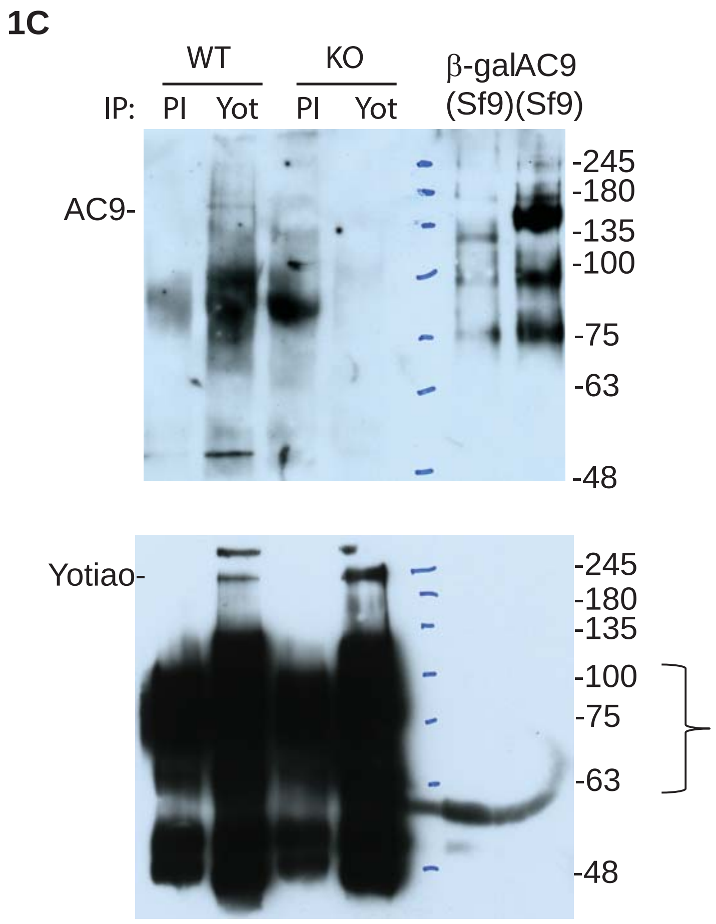

Supplemental Fig 6. Full length western blots, cont.

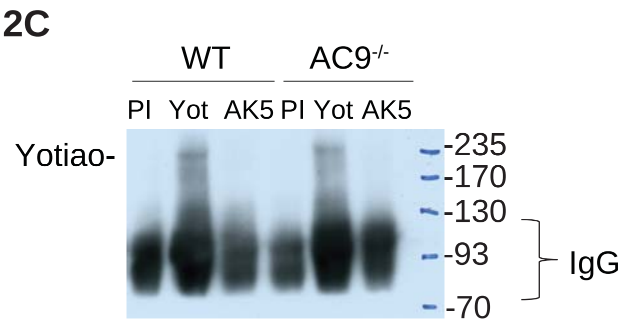

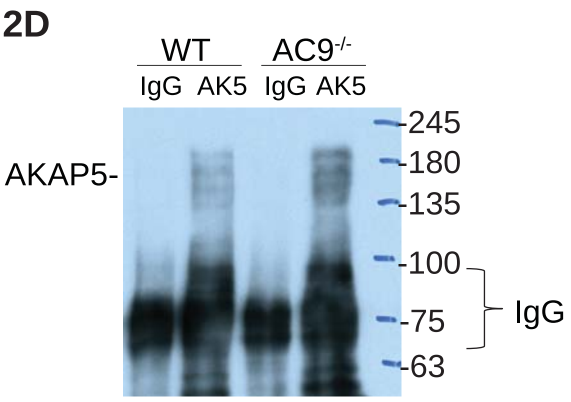

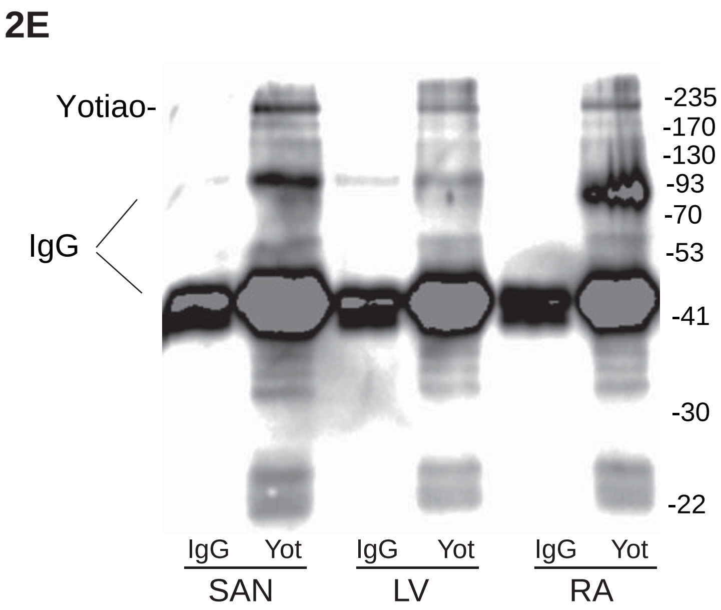

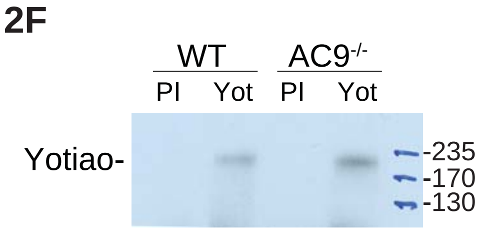

Supplemental Fig 6. Full length western blots, cont.

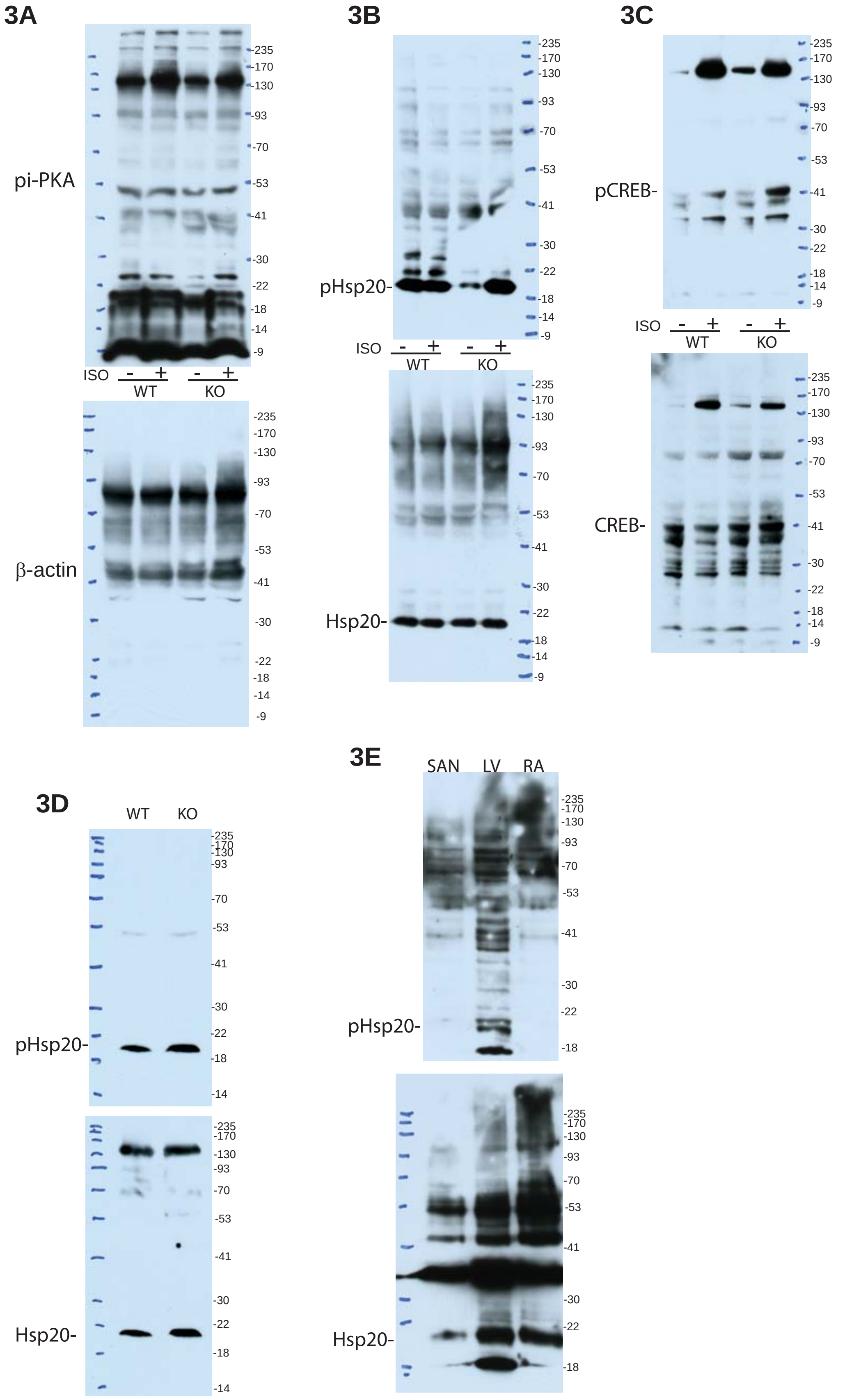

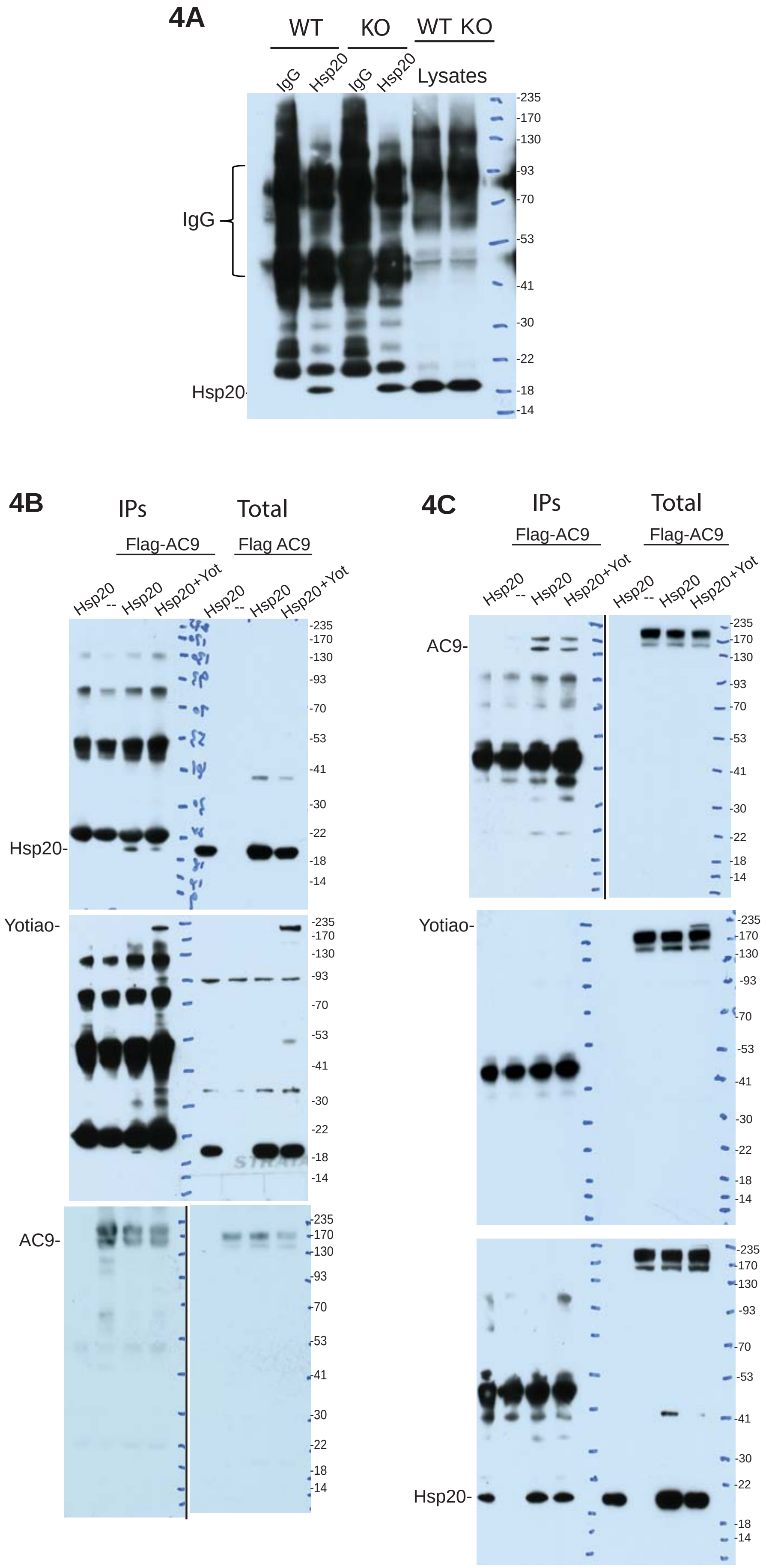
4A
WT
KO
WT KO
IgG
Hsp20
IgG
Hsp20
Lysates
IgG
Hsp20
-235
-170
-130
-93
-70
-53
-41
-30
-22
-18
-14
4B
IPs
Total
Flag-AC9
Flag AC9
Hsp20
--
Hsp20
Hsp20+Yot
Hsp20
--
Hsp20
Hsp20+Yot
Hsp20-
Yotiao-
AC9-
-235
-170
-130
-93
-70
-53
-41
-30
-22
-18
-14
4C
IPs
Total
Flag-AC9
Flag-AC9
Hsp20
--
Hsp20
Hsp20+Yot
Hsp20
--
Hsp20
Hsp20+Yot
AC9-
Yotiao-
Hsp20-
-235
-170
-130
-93
-70
-53
-41
-30
-22
-18
-14

Supplemental Fig 6. Full length western blots, cont.

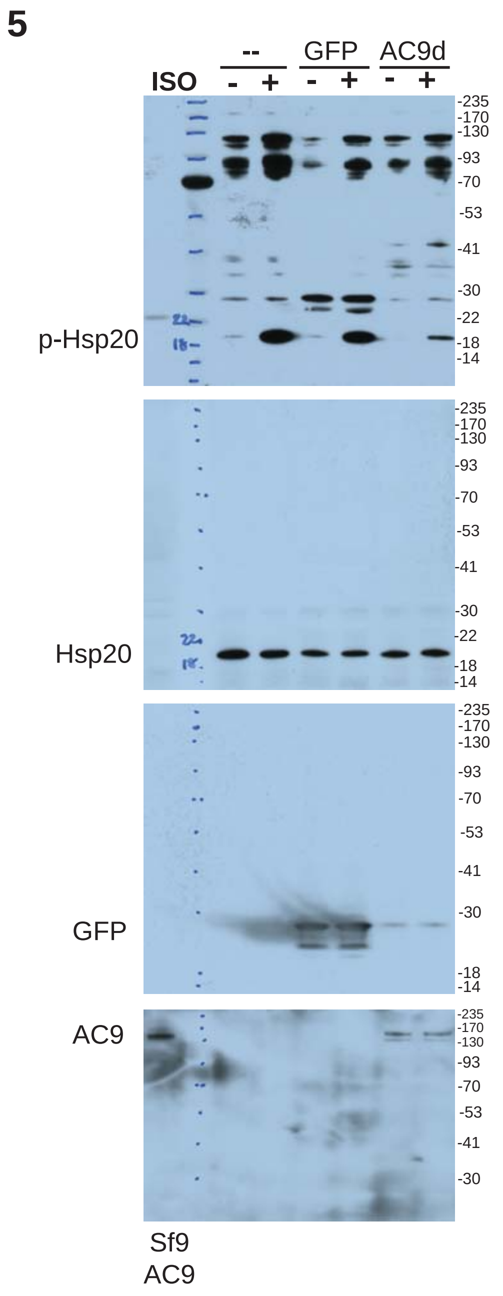

Supplement: Supplementary file 1 — Supplemental material and figures [file 41598_2017_5816_MOESM1_ESM.pdf]
